# Supplementary material for: Combined Use of a Bacterial Consortium and Early-Colonizing Plants as a Treatment for Soil Recovery after Fire: A Model Based on Los Guájares (Granada, Spain) Wildfire
Source: Biology (Basel). 2023 Aug 5;12(8):1093. doi: 10.3390/biology12081093 (PMC10452388; doi:10.3390/biology12081093)
Supplement: Supplementary file 1 [file biology-12-01093-s001.zip › Legends of Supplementary figures.pdf]

Supplementary Figures: Legends

Figure S1. Distribution of culturable population in the samples. The pie chart graph shows the population registered in the mix of ashes (**a**), where green-colored sectors represent Sphingobacterium strains; in red, Acinetobacter calcoaceticus; in golden, Pantoea agglomerans; and in blue, Pseudomonas. The unidentified strains are represented by grey-colored sectors. On the other hand, the bars graph (**b**) shows the amount of colony forming units (CFUs) per mg of sample recorded under each sample condition from three independent replicas. Bars in green correspond to samples from unburnt locations, meanwhile bars in grey correspond to samples from burnt locations. The asterisks represent a statistically significant difference at $p < 0.05$, *, $p < 0.01$, **, and ns stands for statistically non-significant values. Error bars represent s.d.

Figure S2. Species overlapping among burnt and unburnt locations. The analysis of species identified among the isolated strains are showed in the Venn diagrams for roots (**a**) and soil (**b**) samples for the burnt (red-colored bubble) and unburnt locations (green-colored bubble). Size of each studied group are included in graph bars.

Figure S3. Biodiversity indexes. The bar graphs show the calculated indexes of Evenness (E) (**a**), the Shannon-Weiner index (H) (**b**), and the Simpson's index (D) (**c**), including the diversity (1 – D) (**d**) and the reciprocal (1/D) (**e**) under control conditions (green-colored bars) and burnt-mimicking conditions (grey-colored bars) in each microcosm (n = 3). The sets of data were compared using a two-ways ANOVA, where the asterisks represent a statistically significant difference at $p < 0.05$ *, $p < 0.01$ **, $p < 0.001$ ***, and $p < 0.0001$, ****; meanwhile ns stands for groups with no statistical difference respect to the control. Error bars represent s.d.

Figure S4. Petri dish-based screening methods. The images show a representative plate from the test performed in plate for qualitative assessment of the performance of the isolated strains in phosphate solubilization (**a**), where discoloration halos indicate a positive strain; in sulfur oxidation (**b**), where discoloration halos indicate a positive strain; in potassium solubilization (**c**), where yellow halo indicate a positive strain; in nitrogen fixation (**d**), where yellowish halos indicate a positive strain; and in siderophore production (**e**), where again a yellow halo indicate a positive strain. Each strain was tested in triplicate and the halos size was measured in order to compare the relative activity for each trait.

Figure S5. Visual screening of the microcosms. The picture shows the representative microcosms status 40 days after the beginning of the experiment for control and burnt-mimicking conditions. Here, 'Mock' sets stand for the untreated set of microcosms; 'Bb', for microcosms with only *Bituminaria bituminosa* seeds; 'C', for microcosms treated with the bacterial consortium; and 'Bb + C', for the microcosms with both *B. Bituminaria* seeds and treated with the bacterial consortium.

Figure S6. Comparison of *Bituminaria bituminosa* seedlings evolution under each microcosm. The picture shows the representative seedling phenotype that germinated and grown during 40 days in the control and the burnt-mimicking microcosms. Here, 'Bb' labels stand for seedlings from microcosms with only *Bituminaria bituminosa* seeds, and 'Bb + Cons.', for the seedlings from microcosms with both *B. Bituminaria* seeds and treated with the bacterial consortium.
