# Supplementary figures and images for: Combined Use of a Bacterial Consortium and Early-Colonizing Plants as a Treatment for Soil Recovery after Fire: A Model Based on Los Guájares (Granada, Spain) Wildfire
Source: Biology (Basel). 2023 Aug 5;12(8):1093. doi: 10.3390/biology12081093 (PMC10452388; doi:10.3390/biology12081093)

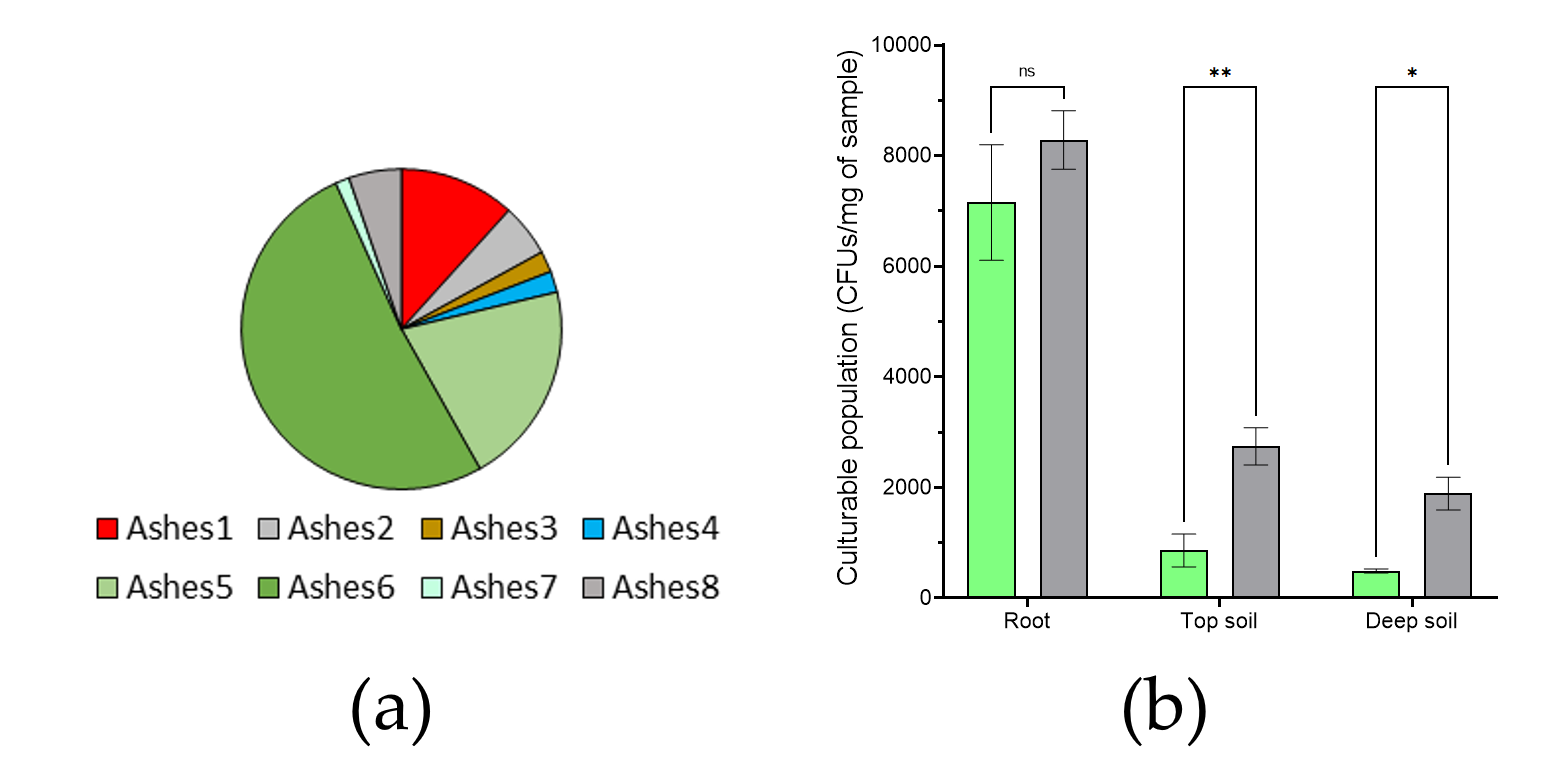

Supplement: Supplementary file 1 [file biology-12-01093-s001.zip › Fig. S1.tiff]

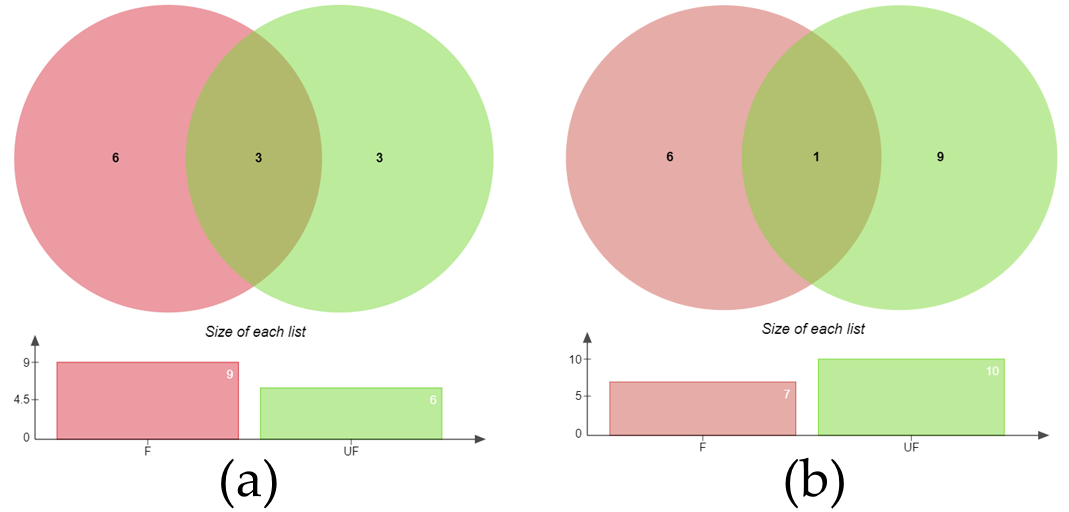

Supplement: Supplementary file 1 [file biology-12-01093-s001.zip › Fig. S2.tiff]

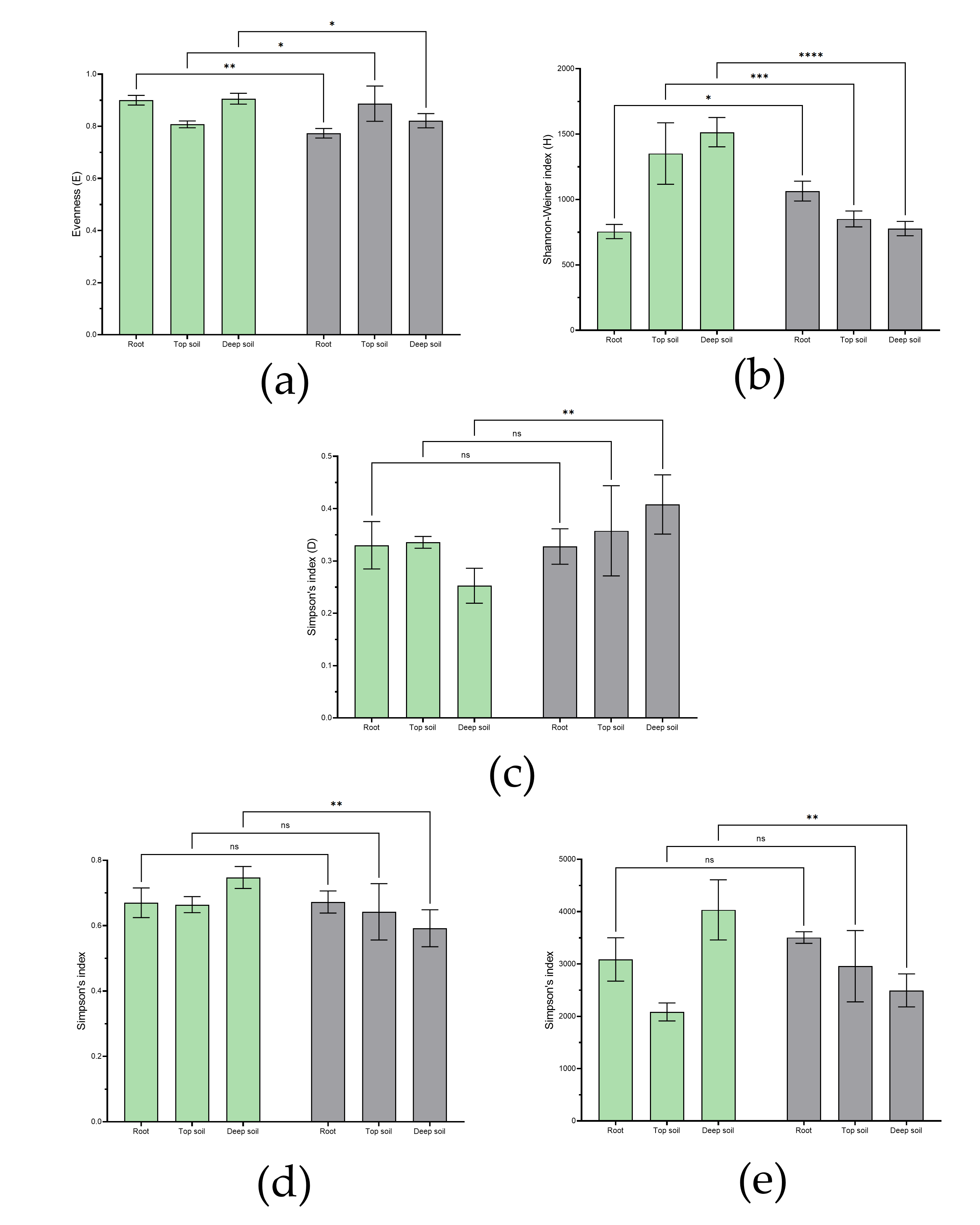

Supplement: Supplementary file 1 [file biology-12-01093-s001.zip › Fig. S3.tiff]

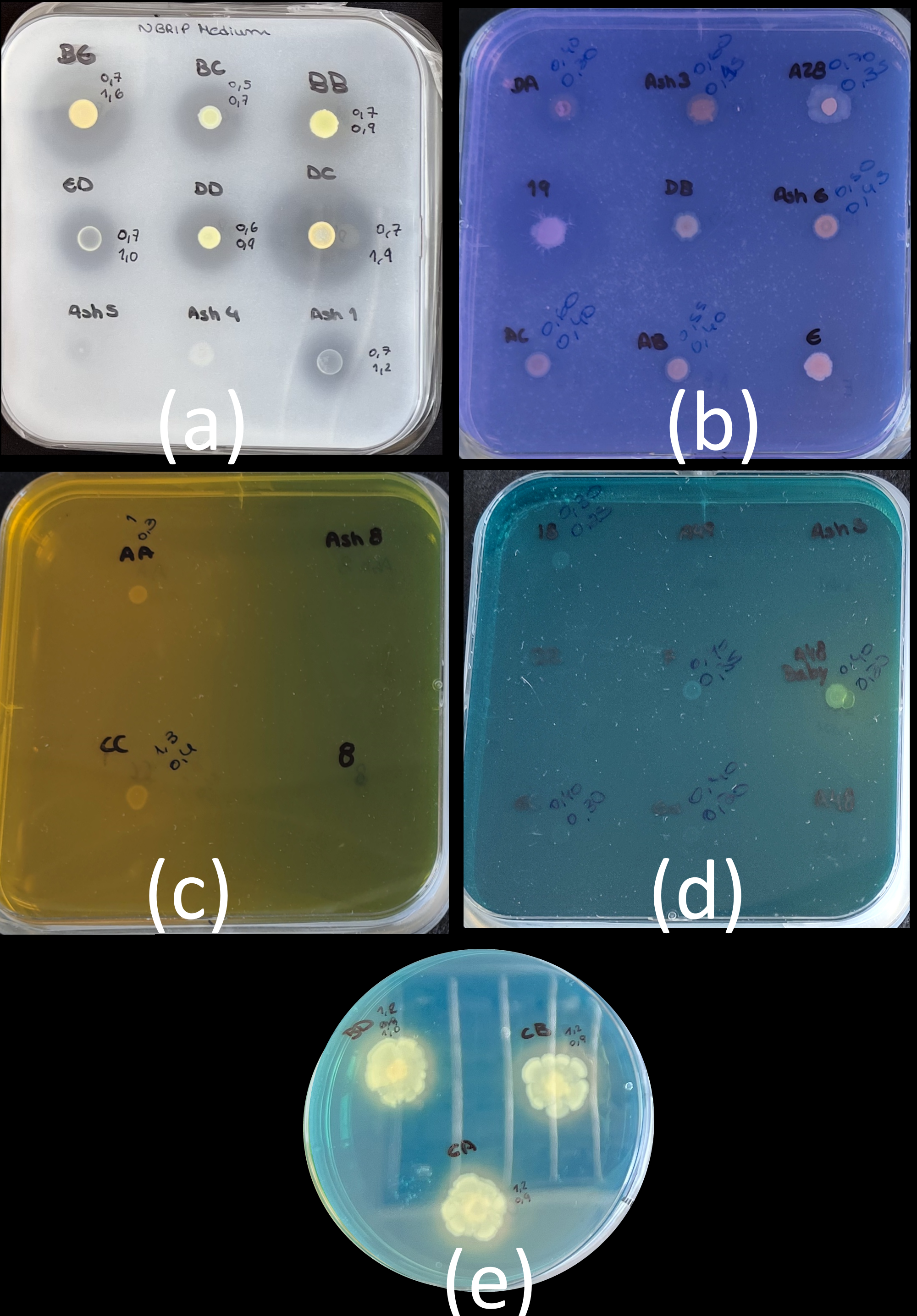

Supplement: Supplementary file 1 [file biology-12-01093-s001.zip › Fig. S4.tiff]

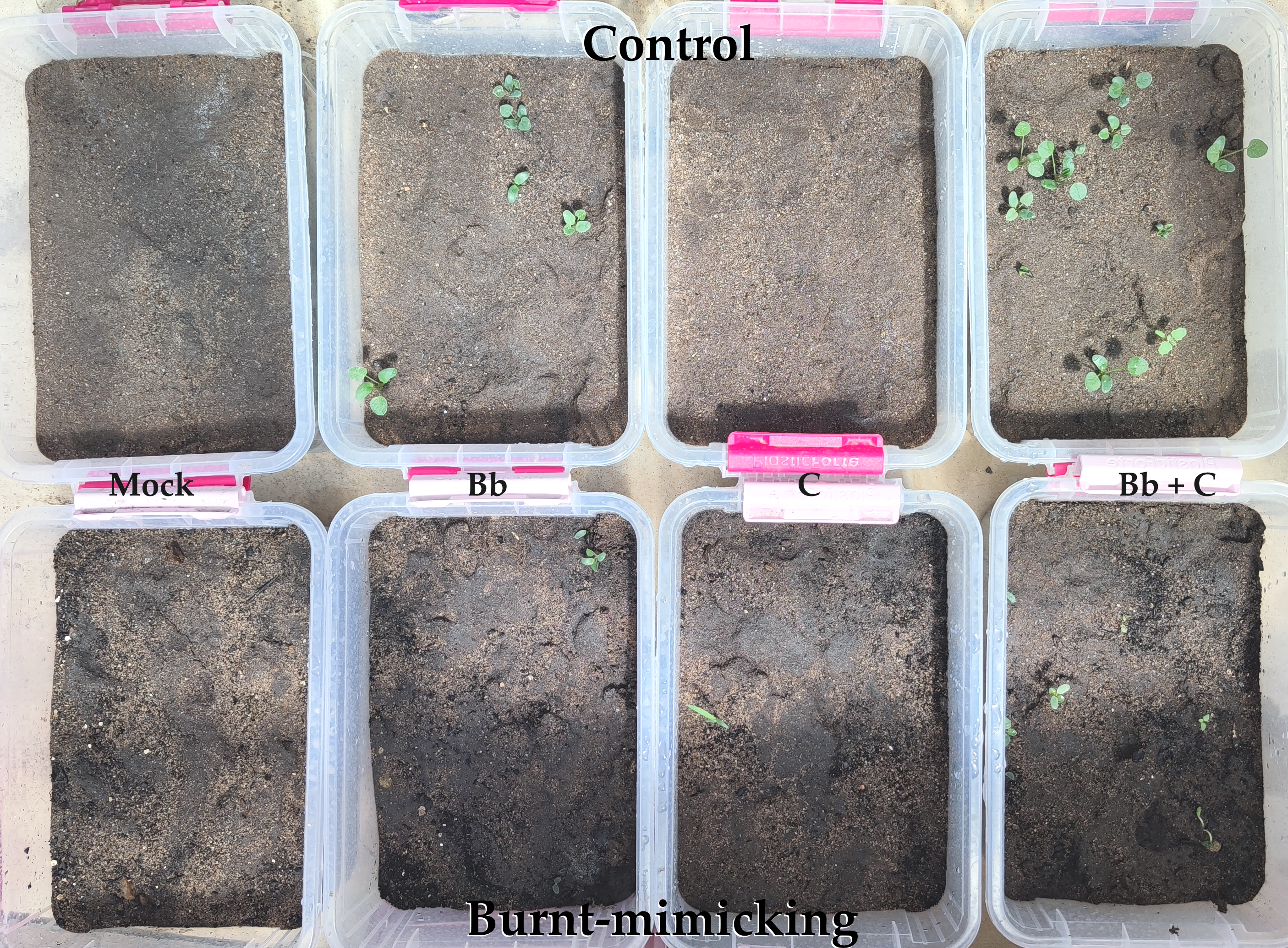

Supplement: Supplementary file 1 [file biology-12-01093-s001.zip › Fig. S5.tiff]

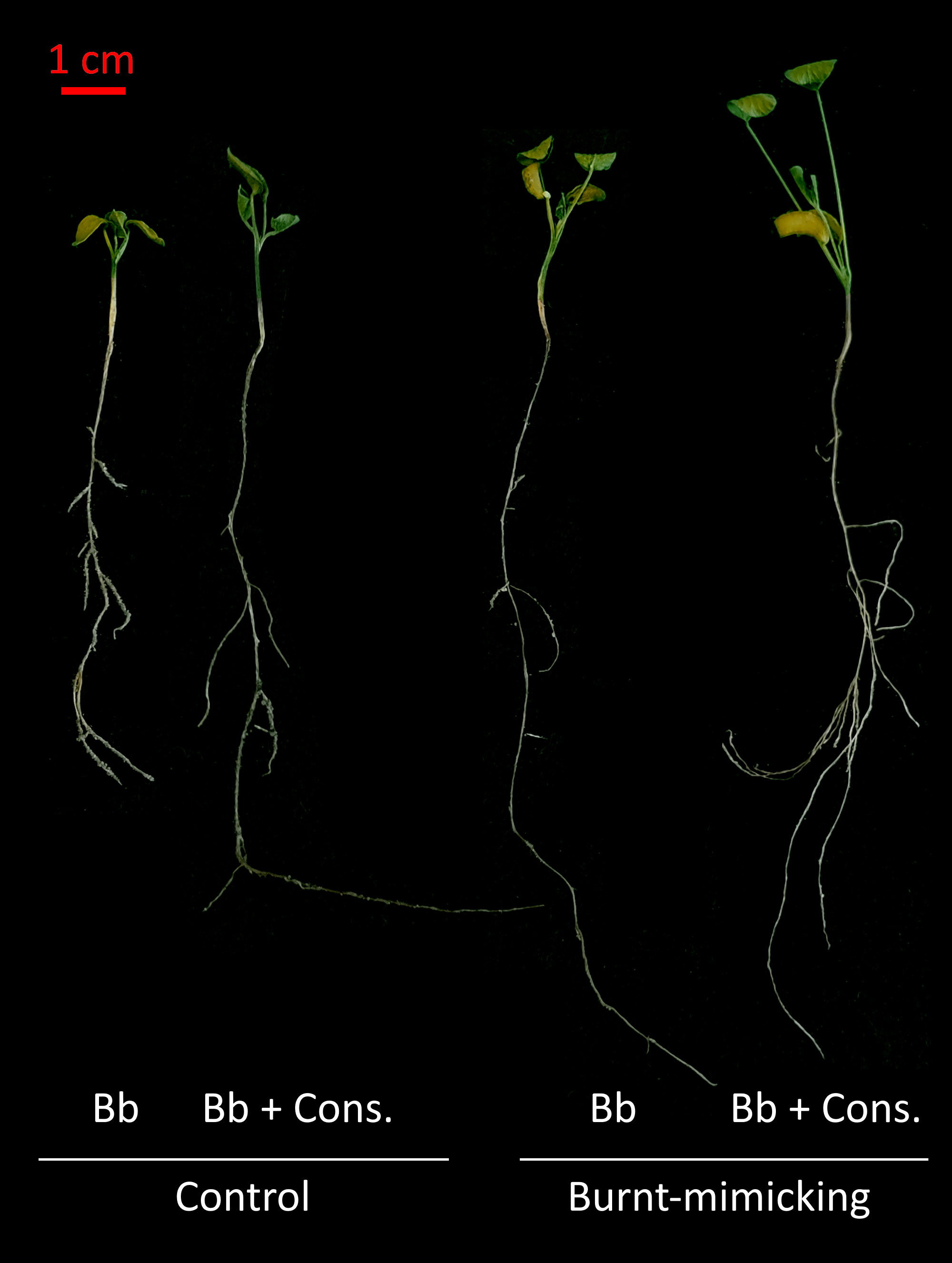

Supplement: Supplementary file 1 [file biology-12-01093-s001.zip › Fig. S6.tiff]
